# Product News

**DOI:** 10.1155/S1463924682000133

**Published:** 1982


Journal of Automatic Chemistry, Volume 4, Number 1 (January–March 1982), pages 45–49

### Product News

### Self-sealing quartz crucible

The FISHER brand® self-sealing crucible makes moisture, volatiles and ash determinations more convenient and repeatable than conventional crucibles and methods. Fisher Scientific say that the crucible can withstand temperatures of up to 1000°C and can be used with a wide variety of samples: coal, coffee, food,

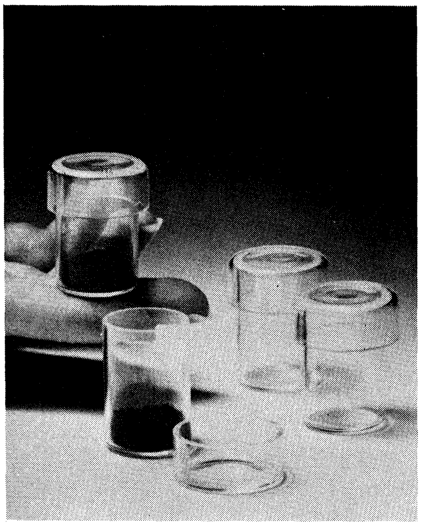

*Self-sealing quartz crucible (Fisher Scientific Co.).*

paper, sugar, tobacco and fertilizers for example, Fisher's crucible features a unique self-sealing lid which forms an air-tight seal around the crucible's ground-glass rim. The lid rises automatically during a volatiles run to expel gasses, then it sinks to restore the seal and form an oxygen-free atmosphere thus preventing sample ashing.

*Further information (ask for Bulletin No. 602) from Fisher Scientific Co., 711 Forbes Avenue, Pittsburgh, PA 15219, USA. Tel.: (412) 562 8546.*

### Gas chromatography

Two companies have sent information about new gas chromatography systems to the *Journal*.

Perkin Elmer have added to their SIGMA range producing a machine which 'features the latest developments in computer-aided chemistry'. The SIGMA 115 provides chromatographic control for two chromatographs and will simultaneously integrate up to four detector amplifier signals. With the necessary accessories SIGMA 115 is a totally automated analytical system, which can be developed to allow real-time display of chromatograms, or display from disc storage. Chromatograms can be overlaid for peak-by-peak comparison, subtracted from one another or manipulated individually.

Techmation are the UK distributors of Carle's 500 CGC which 'uses an advanced microprocessor for precise programmable control of all GC parameters'. The 500 CGC includes three temperature-controlled ovens—one programmable and the other isothermal, a newly designed flame-ionization detector and thermal conductivity detector which allows switching and automatic flow control and monitoring. The package is suitable for conventional or capillary chromatography and can be custom-designed for individual applications.

*Further information on the SIGMA 115 from Perkin-Elmer, Post Office Lane, Beaconsfield, Buckinghamshire HP9 1QA, UK. Tel: 04946 6161; and on the Carle 500 CGC from Techmation Ltd, 58 Edgware Way, Edgware, Middlesex HA8 8JP. Tel.: 01 958 3111.*

### Fluorescence detector

A new fluorescence detector for high-speed liquid chromatography has been announced by Perkin-Elmer. Model LS4 Fluorescence Spectrometer combines their LS series with an optical system using a 3 $\mu$l illuminated volume flow cell.

The excitation monochromator is optimized at 250 nm allowing operation down to 200 nm. The monochromators cover the range 200–800 nm and are stepper-motor driven with digital display of wavelength. A wavelength program mode enables up to 15 pairs of excitation and emission wavelengths to be changed with respect to time thereby optimizing sensitivity and specificity through the chromatogram.

Full computer control and data handling is possible with the company's Model 3600 Data Station and RS232C interface.

*Further information from Perkin-Elmer Ltd, Post Office Lane, Beaconsfield, Buckinghamshire HP9 1QA, UK. Tel.: 04946 6161.*

### Coal analysis

Nova-Coal 1 (Standard Instrumentation Inc.) is a 'computerized control lab. system' which, when interfaced with existing equipment, provides direct readings from a digital balance, monitors a moisture oven and ash-fusion furnace and controls ash and volatile furnaces and an adiabatic calorimeter. Nova-Coal 1 uses a DG MicroNova computer to automate tests on moisture content of coal, ash, BTU, sulphur, volatile matter, FSI, and ash-fusion temperatures. Installing Nova-Coal 1 can reduce a coal laboratory's costs by making operators more

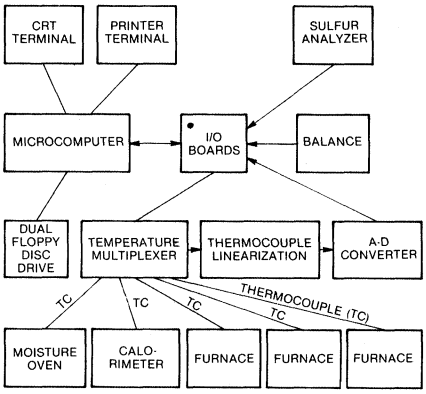

*Nova-Coal 1: hardware block diagram. The microcomputer with 32k-word core memory interfaces to laboratory instruments through I/O boards and temperature multiplexer. Each I/O board has 16 input and 16 output lines. MUX routes thermocouple signals through linearization network and then to ADC. Since the calorimeter requires no linearization, it is actually read directly to the ADC (circuit not shown on diagram). (Standard Instrumentation Inc.)*

productive (by 50 to 100% depending on the operator and condition and type of laboratory equipment), using clerks to analyse coal rather than chemists, reducing equipment costs (no need to buy furnace controllers and digital read-outs) and eliminating manual calculation. The computer can also be used for book-keeping, accounting, payroll, inventory control and engineering calculations.

Nova-Coal 1 improves test accuracy because balance weights are read automatically, thus removing the chance of human error in transcription. It also improves test repeatability and provides test results on demand. In the event of a computer malfunction, the controls can be operated manually.

*Further information from: Standard Instrumentation Inc., 3322 Pennsylvania Avenue, Charleston, WV25302, USA. Tel.: (304) 345 4727.*

### Low-cost check for gas detectors

For personnel working in potentially explosive or inflammable areas Crowcon have designed a low-cost sensitivity test kit for gas detectors. The kit is intended to be used to check Crowcon's own Gasalarm 76GA but can be adapted to other alarm systems. The kit contains an aerosol can filled with methane (2·5% in air), a fine-control valve, tubing and an O-ring. Gas alarms are checked by plugging the kit into the detector: if the meter indicates between 45% and 55% L.E.L. for methane, it is operating within its sensitivity range. Crowcon can give approximate percentage readings for other gasses. The kit is a cheap, quick and simple way of carrying out daily or prior-to-use tests for safety.

*It costs £15·00 and is available from Crowcon Instruments Ltd, Temple Road, Cowley, Oxford, UK. Tel.: 0865 776707.*

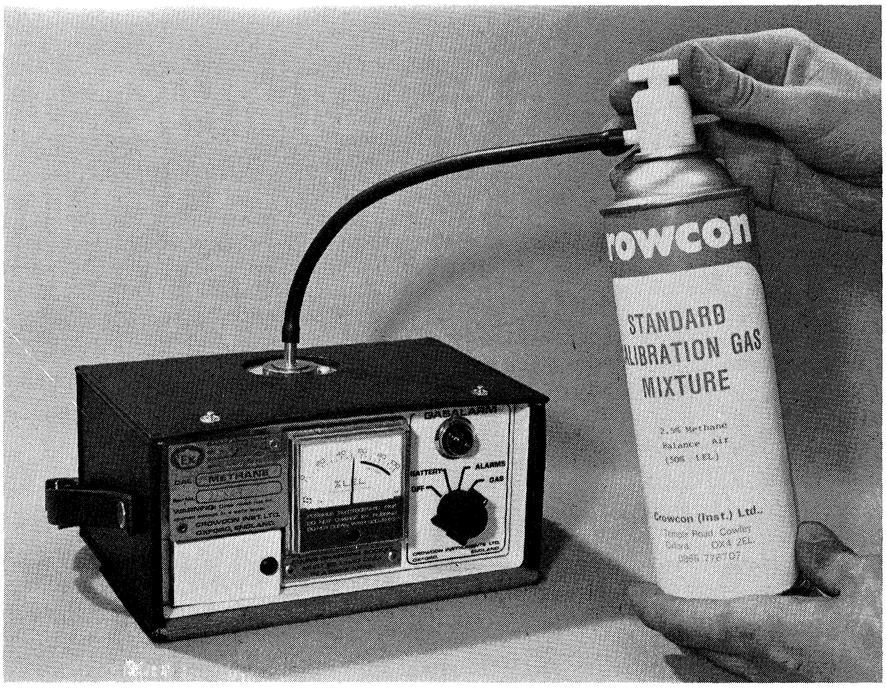

*The new test kit being used on one of Crowcon's 'Gasalarms'. (Crowcon Instruments Ltd.)*

### 'Instrument News'

Perkin-Elmer's annual *Instrument News* takes computer-aided chemistry as its theme for 1981. The magazine is a catalogue of the company's products and services and includes information on laboratory information systems, and on machines for atomic spectroscopy, environmental sampling, gas chromatography, infra-red and ultra-violet measurement, liquid chromatography, thermal analysis and polarimetry.

*'Instrument News' is available from Perkin-Elmer Ltd, Post Office Lane, Beaconsfield, Buckinghamshire HP9 1QA, UK. Tel.: 04946 6161.*

### Programmable A-D converter

Dyson Instruments have designed a precision analogue-to-digital converter to take the signals a scientist normally displays on a strip-chart recorder, digitize them and pass them into a microcomputer.

Construction is based on voltage to frequency conversion and its special features are a high accuracy and resolution making it ideally suitable for many demanding laboratory roles, such as chromatography and spectroscopy. Input range is $-5$mV to $+10$ volts DC (self-ranging). Resolution is one count per $\mu$V input in the critical region of $\pm 5$ mV, and the crystal-controlled data-slice rate can be manually set at 10, 100 or 1000 ms.

*Further information from Dyson Instruments, Sunderland House, Station Road, Hetton, Houghton-le-Spring, Tyne & Wear DH5 0AT, UK. Tel.: 0783 260452.*

### New pH electrode

The first protein-resisting laboratory pH electrode, which will solve the expensive and time-consuming problem of protein-coating, has been announced by Russell pH Ltd. Any electrode used where proteins are present is likely to become coated. Russell have tested their new electrode's performance in strong protein solution and it gave accurate pH readings over the six months of the experiment. The standard electrode tested in parallel with it showed symptoms of protein coating after two weeks.

*Full details from: Russell pH Ltd, Station Road, Auchtermuchty, Fife KY14 7DP, UK. Tel.: 03372 480.*

### Haematology

The H6000, Technicon's fully automated routine haematology system, now analyses 90 samples per hour. The old H6000s run 60 samples/h. Tests include complete blood count, distribution widths and cell size histograms for red cells and platelets, total and differential white cell counts. H6000 systems already in use can be 'retrofitted' up to the 90 samples/h specification. New H6000/90 machines will be available early in 1982.

*Further information from: Technicon Instruments Company Ltd, Evans House, Hamilton Close, Basingstoke, Hants RG21 2YE, UK. Tel.: 0256 29181.*

### Electrophoretic blotting

Two new products have been added to Bio-Rad Laboratories' range of electrophoresis equipment. Their Trans-Blot Cell is designed for quick electrophoretic blotting of DNA, RNA and protein. The instrument uses platinum electrodes, has triple gel capacity and optional internal cooling. Users can transfer electrophoretically separated DNA, RNA or protein from the gel to an immobilizing matrix in 30 min. Once transferred, the bands are readily accessible for auto-radiography, ELISA or fluorescent detection and for preparative elution. The cell holds up to three gels which can be as large as 20 × 16 cm, and requires 2–3 l of buffer.

The Model 160/1.6 Power Supply is designed to meet the power requirements of all electrophoretic transfer techniques. The Model 160/1.6 can produce an output of up to 1·6 amps, which is essential for the maintenance of a uniform electric field across the cross-sectional area of the gel. It is designed for use with the Trans-Blot Cell, but will also be useful for DNA chemists involved with submarine agarose bridge horizontal gels.

The Trans-Blot Cell and Model 160/1.6 Power Supply are available either individually, or combined as a system.

*Further information from David Forrester, Bio-Rad Laboratories Ltd, Caxton Way, Holywell Industrial Estate, Watford, Hertfordshire WD1 8RP, UK. Tel.: 0923 40322.*

### Blood-banking

Kontron Analytical have announced two new machines for blood-banking. The Groupamatic 2000 is a blood-grouper for small centres; it can be used either as a nine-channel system grouping 240 samples/h or as a 18-channel system grouping 120 samples/h. Groupamatic 2000 analyses using a discrete method, each test is carried out in an individual cuvette and result interpretation is automatic. Their second new product, called Transfudata, is a data-processing package which can computerize many of the areas in blood-transfusion centres. Transfudata is available either as a complete package or as a software suite for centres already using appropriate hardware.

*Further information from Jim Bradley, Kontron Instruments Ltd, Kontron House, Campfield Road, St. Albans, Hertfordshire, UK. Tel.: 0727 66222.*

### Process instrumentation school

The Institute of Measurement and Control are holding their Eighth School on Process Analytical Instrumentation at the University of Warwick from 22 to 26 March 1982. The School will be of help to those new to the instrument field, to those with some experience of instrumentation who want to broaden their knowledge, and to contractors and managers who are indirectly concerned with analytical instruments. 28 lectures will be given on, for example, process analytical system design, sampling, electronics, electrochemistry, spectroscopy, mass spectrometry, gas chromatography, oil-refinery analysers, microprocessors. Participants will also take part in project work in the evenings.

*Further information from the Deputy Secretary, Institute of Measurement and Control, 20 Peel Street, London W8 7PD. Tel.: 01 727 0083.*

### Mositure measurement

Anachem Ltd are offering a contract application service for the determination of moisture in samples of liquids, gasses and solids. The service will be of help to people who only have occasional samples to analyse, or particularly awkward ones and are unable to justify buying their own equipment. Anachem's technicians use Mitsubishi's automatic moisture meters and vaporizers; the company also sell Mitsubishi's instruments. Customers using the application service are supplied with confidential reports and full method development details. A standard fee is charged for the initial method development and thereafter a charge is made for each sample analysed.

Anachem also run one-day seminar/workshops on moisture measurement using Mitsubishi meters and vaporizers.

*Further details on the application service and seminars from Anachem Ltd, 15 Power Court, Luton, Bedfordshire LU1 3JJ. Tel.: 0582 35252.*

### MICROLAB 2000

A universal computing sample-processing system called MICROLAB 2000 has been launched by Hamilton of Switzerland. MICROLAB 2000 handles all the liquid-transfer operations associated with a clinical laboratory, including pipetting and dilution of samples. The instrument works with samples from 5 $\mu$l and reagent volumes up to 1 ml; it can dispense into most analyser tubes. The sample rack holds up to 500 tubes which can be thermostatted and sealed, up to 10 reagents are held in an adjacent rack containing four individually controlled magnetic stirrers. The unit has a built-in wash station to minimize carry-over, and an integrated liquid detector which automatically sets the depth of immersion of the pick-up tip. It uses a sip-and-dip method of transfer and loading of reagents and samples is very quick—each of the 500 samples can be transferred in under a second. Information is fed into the computer via a hand-held bar-code reader. MICROLAB 2000 uses the MICROLAB M diluter/dispenser and Hamilton gas-tight Microliter syringes.

*Further information from Hamilton Bonaduz AG, PO Box 26, CH 7402 Bonaduz, Switzerland. Tel.: 081 811939.*

### 'Plasmaline'

The latest issue of *Plasmaline*, a quarterly newsletter for users of DC plasma-emission spectrometers, is available from SpectraMetrics Inc. or from Techmation. Volume 2, Number 2 contains papers on the analysis of coal and on the determination of arsenic and selemium in a high-salt nickel matrix. It has an 'Applications Hints' section which provides information on analysis of complex matrices, such as geological or waste-water samples.

*'Plasmaline' is free from Spectrametrics Inc., 204 Andover Street, Andover, Massachusetts 01810, USA; or from Techmation Ltd, 58 Edgware Way, Edgware, Middlesex HA8 8JP, UK. Tel.: 01 958 3111.*

### Acid rain

The US Environmental Protection Agency (EPA) recommends ion chromatography (IC) for acid-rain monitoring and research stations. Dionex have published an assessment of the suitability of their fast-run anion columns for the determination of anions in acid rain. Using the EPA's method for measuring $Cl^-$, $PO_4^{3-}$, $NO_3^-$, and $SO_4^{2-}$ in rain-water, Dionex analysed NBS synthetic acid-rain standards. Their fast-run columns are shown to offer a much faster analysis and greater sensitivity than standard IC anion columns.

*The full report is available from the Dionex Corporation, 1228 Titan Way, Sunnyvale, California 94086, USA. Tel.: (408) 737 0700; or from Dionex (UK) Ltd, First Floor, The Parade, Frimley, Camberley, Surrey, UK. Tel.: 0276 29771.*

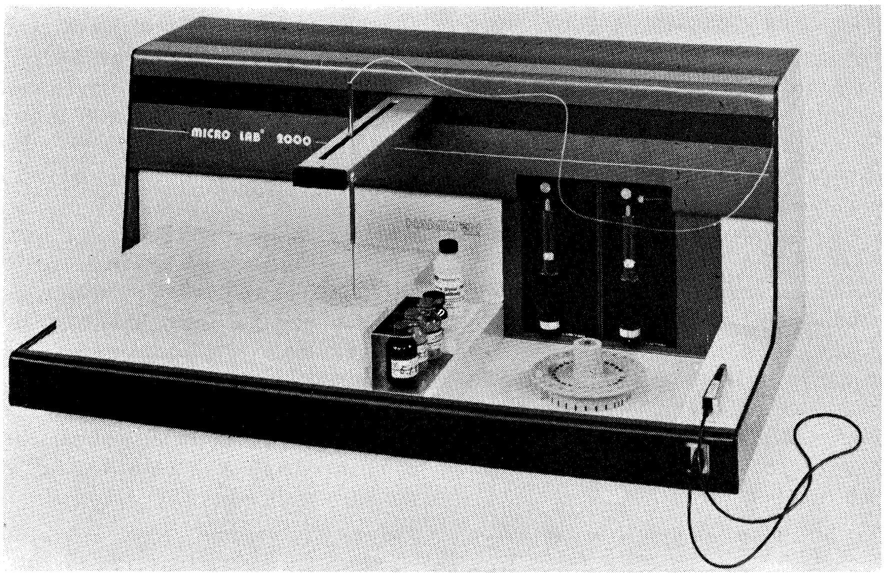

*MICROLAB 2000—a universal computing sample-processing system. (Hamilton Bonaduz AG, Switzerland.)*

### High-risk pregnancies

Byk-Mallinckrodt have developed a radioimmunoassay kit to monitor high-risk pregnancies. The kit measures human placental lactogen (HPL) levels in patient serum—HPL levels which are low relative to the progress of the pregnancy indicate a degree of foetal stress which could manifest itself either by premature abortion or a stillbirth. The kit is based on the double-antibody separation principle.

HPL in aliquots of patient sera and I-125 labelled HPL are allowed to compete for binding sites on a specific antibody. This is raised against human HPL in sheep. The greater the level of HPL in the patient sera, the less I-125 HPL will be bound to the antibody. The I-125 HPL/antibody complex is then precipitated out of solution using a second antibody in PEG (Polyethyleneglycol) solution. After centrifugation is discarded and the precipitate is counted to determine the amount of I-125 HPL bound to the antibody. A standard curve is prepared and the patient's HPL concentration in serum is evaluated by comparing the serum sample counts to the standard curve.

*Further information from Peter Walton, Byk-Mallinckrodt (UK) Ltd, 129G Building 521, Heathrow Airport, Hounslow, Middlesex TW6 3TH, UK. Tel.: 01 897 2425.*

### CRT testing

A precision CRT test set is available from Bertan Associates. Model CRT-30 is a three output voltage power supply which is specifically configured as a cathode ray tube test set. The instrument includes all the controls, monitors and protective features which are necessary to the operation and evaluation of CRT performance.

Three independent precision controls provide fine adjustment of the anode, focus and G2 outputs. The voltage and current of each of the outputs can be monitored on the front panel meters. Options are available to expand the capability of the standard CRT-30. They include G1 and filament outputs, remote programming and monitoring (both digital and analogue) as well as dynamic focus capability. All outputs are provided with 0·01% line and load regulation and with ripple of less than 0·01% pk-pk at maximum output. The temperature coefficient is better than 100 ppm/°C.

*Further information from Howard Rachlin, Bertan Associates, Inc., 3 Aerial Way, Syosset, New York 11791. Tel.: (516) 433 3110.*

### Counting on computers

A high-speed digital counter with a computer interface, specifically designed for nuclear radiation or other pulse-counting applications, has been developed by ESI-Panax. The instrument, SCALINK 4, can be connected to many desk-top computers, calculators and GPIB controllers using a standard IEEE-488 data bus. In a typical application using a Commodore PET the counting interval can be selected from a 'menu' displayed on the screen, and each result processed or stored as required. Alternatively, the computer can determine the counting interval automatically based on the number of counts received and the required statistical accuracy. SCALINK 4 is fully addressable, allowing up to 16 modules to operate simultaneously with one computer. Programming is simple using BASIC 'INPUT' statements to read the contents of each scaler, the result appears as a numeric variable. The unit requires a single 5-volt supply and is fully compatible with other NIM modules.

*Further information from AID Scientific Ltd, Canford Works, Wallisdown Road, Bournemouth, Dorset BH11 8QW, UK. Tel.: 0202 524152.*

### Bendix UK

The Bendix Corporation have opened a new office in the UK. The Industrial Instruments Division, at Milton Keynes, will provide sales, service, administration and technical support for the products manufactured by the Corporation's American Environmental and Process Instrument Division—process gas chromatographs, source and ambient air quality monitors and health and safety-related instrumentation.

*Further information from Bendix Ltd, Industrial Instruments Division, 72 Tanners Drive, Blakelands, Milton Keynes MK14 5BP, UK. Tel.: 0908 614343.*

### Micro interfaces

A range of microcomputer interfaces, which can be used with most micros—PETs, Apples and Intertec Superbrains for example—has been produced by Anaspec. Each interface consists of a computer module and an input/output module; they come complete with a manual and the necessary cables. The company offers four varieties of computer module and 10 types of I/O module.

*Further information from: Anaspec Research Laboratories Ltd, Pearl House, Bartholomew Street, Newbury, Berkshire, UK. Tel.: 0635 44329*

### Tunable dye laser

Model 2100 Dyescan tunable dye-laser system is the first to incorporate a nitrogen pump laser, dye-laser assembly and complete microprocessor control into one cabinet. The system is intended for spectroscopic applications which require a precision, narrow line-width, scannable, UV-visible source. The nitrogen pump laser eliminates the need for a vacuum system. The dye-laser provides output pulses of 1 ns duration and 25 kw peak power; line-width is 0·03 nm at 590 nm. Model 2100 Dyescan is under complete microprocessor control. Tuning range is between 360 and 800 nm and a frequency-doubling accessory will provide access to the UV region of the spectrum. Scans between operator-specified limits can be linear either in wavelength or energy units. A unique optogalvanic calibration technique provides rapid, highly accurate wavelength calibration, while the dye-laser power is continuously monitored. All parameters and commands are entered through a simple keyboard.

*The Model 2100 Dyescan is available from EG+G Princeton Applied Research , PO Box 2565, Princeton, New Jersey 08540, USA. Tel.: (609) 452 2111.*

### Filter fluorometer

The American Research Products Corporation's Fluoro-Tec Filter Fluorometer, with digital photomultiplier microphotometer module, has been introduced into Europe by Plasma-Therm Ltd. The instrument is designed for both cuvette operation and continuous-flow analysis. For HPLC and flow-injection applications the Fluoro-Tec offers several quick-change flow-cell configurations and cuvette adapters with slip-in exchange features. Accessories are also available to allow the Fluoro-Tec to be used for chemiluminesence and biluminesence. In addition to the four-watt mercury vapour light-source and 931-B photomultiplier detector supplied as standard, a Xenon light-source and a S-20 response photomultiplier tube are available. This extends the range and sensitivity of the instrument. The instrument offers unparallelled stability and sensitivity at a low cost, and is an easy to operate, multiple-purpose filter fluorometer.

*Further information from Plasma-Therm Ltd, 6 Station Road, London SE20 7BQ. Tel.: 01 778 6798; or from the American Research Products Corporation, 4232A Howard Avenue, Kensington, Maryland 20795, USA. Tel.: (301) 946 5530.*

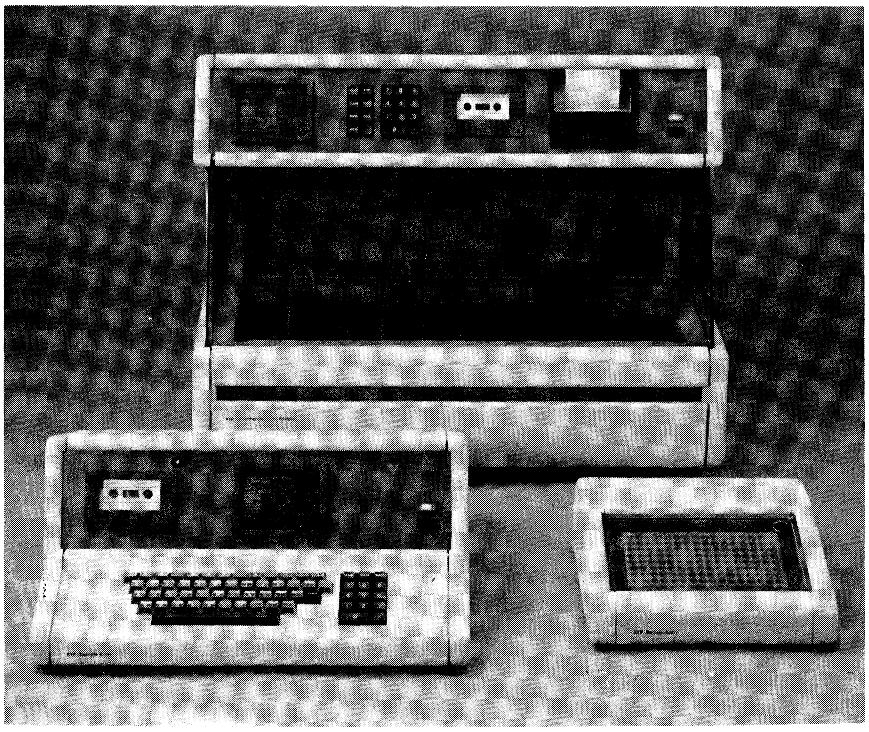

*Vitatron's XYP spectrophotometric analysing system. (Vitatron Scientific B.V., The Netherlands.)*

### Automated spectrophotometry

Vitatron's new automated spectrophotometry system for clinical chemistry laboratories was designed to be cost-effective, simple, versatile, reliable, to provide continous sample control, and to be easy to maintain. The manufacturer has called it the Vitatron XYP, because it incorporates a patient/test matrix and has sample trays which allow each sample to be identified by its X and Y co-ordinates—XY-Processing. The patient/test matrix means that there is continuous identification of, and instantaneous access to, all the samples in a test batch.

A sample tray takes up to 128 samples and each sample can be subjected to 46 pre-programmed tests (more can be added) within 10 different profiles. Patient data and tests required are entered using a sample entry station and are recorded on a digital compact cassette. The operator is told where the samples should be placed in the tray; the XYP works out an optimum test sequence taking into account facts like incubation times.

The instrument is patient-oriented and its controlled sample handling, fixed sample location and electronic detection ensures correct patient identification throughout all procedures. Test results are stored and automatically grouped per patient.

The machine is very cost effective. Firstly, it is competitively priced for an instrument capable of providing up to 480 results/h. Second it reduces the cost of disposables—the 128-position trays are much cheaper than individual containers. Third it saves on reagents—the reaction mixture is 250 $\mu$l; moreover it is an open system so users can shop around for reagents. And, fourthly, because it is automated to such a great degree and is ergonomically designed, it saves operator's time.

*A brochure describing the XYP can be obtained from Vitatron Scientific B.V., PO Box 100, 6950 AC Dieren, The Netherlands. Tel.: 08330 19010.*

### ACA improvement and user training

Du Pont's microprocessor-controlled Automatic Clinical Analyser (ACA) can now perform 42 tests on blood and serum, and has a virtually unlimited channel capacity. The company are offering a week-long training course for ACA users. The course is run in Geneva, 40 are available each year and teaching can be in English, German, French or Italian.

Du Pont are researching further improvements of their ACA and expect shortly to have a system capable of performing 55 tests. The 13 new tests will cover haematology, serology, immunology and therapeutic drug monitoring.

*Details of the ACA and training course from: Du Pont de Nemours International S.A., P.O. Box, CH 1211 Geneva 24, Switzerland. Tel.: 022 378111.*

### Particle sizing

Malvern Instruments have recently introduced a new particle sizer into the UK market. The Autosizer measures size between 10 and 300 nm and diffusion coefficient. The machine has repeat-measure facilities so it can be used for real-time study of particle interaction—for measuring agglomeration and the effects of additives and pollutants.

*Further information from: Malvern Instruments Ltd, Spring Lane, Malvern, Worcestershire WR14 1AL, UK. Tel.: 06845 3531.*

